# Patients responses to diagnoses of mental disorders: Development and validation of a reliable self‐report measure

**DOI:** 10.1002/mpr.1854

**Published:** 2020-09-12

**Authors:** Thomas Schnell, Anja Kehring, Steffen Moritz, Olaf Morgenroth

**Affiliations:** ^1^ Medical School Hamburg University of Applied Sciences and Medical University Hamburg Germany; ^2^ Department of Psychiatry and Psychotherapy University Medical Center Hamburg‐Eppendorf Hamburg Germany

**Keywords:** coping, empowerment, evaluation, questionnaire, self‐stigmatization

## Abstract

**Objectives:**

Psychiatric patients are regularly informed about diagnoses. Treatment guidelines assume that informing patients fosters functional coping processes, but few research exists on how patients respond. Thus, the objective was to develop a standardized self‐report measure to assess patients reactions to diagnoses.

**Methods:**

Fifty nine items were generated based on a qualitative study. The process of item selection and determination of the factor structure were performed on a sample of 252 patients: Results of an explorative factor analysis with a randomly split sub‐sample 1 were cross‐validated by confirmatory factor analysis on sub‐sample 2. The revised 26‐item instrument was revaluated using data from an independent sample of 1.271 patients with different diagnoses.

**Results:**

Three functional and three dysfunctional processing styles emerged from the analyses and provided good model fit in the revaluation study (TLI = 0.935; CFI = 0.943; RMSEA = 0.051; SRMR = 0.048). Variance‐analytical calculations and post hoc analyses revealed significant differences among diagnoses with regard to coping styles, such as schizophrenia was associated with self‐stigmatization and anorexia nervosa showed pronounced over‐identification. Overall, various diagnosis‐dependent specifics were found.

**Conclusions:**

As patients reactions to diagnoses vary substantially, their formation, impact on treatment and overall cause should be investigated in further studies.

## 1 INTRODUCTION

The science philosopher Ian Hacking defined the ‘looping effect’, stating that the communication of diagnosis changes the patient's self‐perception, behaviour and finally the symptoms of the diagnosis themselves (Hacking, 2007). The question is whether patients change for the better or worse once informed about (alleged) diagnoses. With regard to psychological adaptation processes, a global distinction can be made between approach and avoidance motivation. Accordingly, 3 decades ago Roth and Cohen (1986) proposed a concept for coping with diseases based on functional approach coping and dysfunctional avoidance coping. Avoidance impedes functional adaptation of the individual, and approach coping mobilizes resources to ensure successful adaptation to new situations (Fitzpatrick & Stalikas, 2008). Accordingly, approach behaviour empowers patients to functionally cope with diseases (Kramer, Caspar, & Drapeau, 2013).

Empowerment in terms of functional coping is the key objective of the concept of shared decision‐making (SDM; Elwyn et al., 2012). SDM aims at a transparent and open communication with regard to the disease and its treatment. It thus influences how patients perceive their diagnosis and empowers them to make informed decisions (Samalin et al., 2018). Research indicates that SDM has implications for the acceptance of a diagnosis in patients and the further adherence to treatment (Papageorgiou, Loke, & Fromage, 2017; Samalin et al., 2018). Empowerment through SDM also implies that patients develop hope for a satisfactory life despite their diagnosis, and this was associated with successful recovery in a study (Zhang, Mak, & Chan, 2017). In this sense, empowerment as coping corresponds with a well established common factor of psychotherapy according to Grawe (2004): the activation of individual psychological resources. SDM was also explicitly included in the recent revised German S3 treatment guidelines for schizophrenia, aiming to empower patients with a disease that often causes severe despair and dysfunctional coping (Hasan et al., 2020).

Such dysfunctional coping instead of empowerment is described in particular within stigma research, indicating that some diagnoses more than others can trigger a dysfunctional self‐stigmatization, by transferring the public stigma to one's own person (Bravo‐Mehmedbašić & Kučukalić, 2017; Frischknecht, 2017). Especially, if a mental disorder is not associated with hope for recovery, but experienced as a threat to basic needs, a dysfunctional self‐stigmatisation can result (Zhang et al., 2017). This is associated with poor recovery and an overall negative therapy outcome in various psychiatric patients (Kalisova, Michalec, Hadjipapanicolaou, & Raboch, 2018), especially in schizophrenia (Häfner, 2017). Further influencing factors of self‐stigmatization are younger age (Werner, Aviv, & Barak, 2008), unemployment and the number of episodes of mental disorders (Adewuya, Owoeye, Erinfolami, & Ola, 2011).

In addition to self‐stigmatization, another dysfunctional way of dealing with a diagnosis is described as a problematic fixation on psychopathology/diagnosis in the sense of an over‐identification (Kelly & Carter, 2014; Sunkel, 2015). However, very few studies have addressed this issue. Sunkel (2015) shows in a qualitative study that patients with anorexia nervosa (AN), especially when active in so‐called pro‐ANA forums, present dysfunctional over‐identification. According to the author, the diagnosis is seen as a lifestyle rather than a problem. This over‐identification was also associated with poor treatment outcome (Sunkel, 2015). Also, Kelly and Carter (2014) demonstrated that patients with AN more than other psychotherapy patients exhibit a dysfunctional fixation on psychopathology in the sense of over‐identification. The authors discuss this fixation as a negative dimension of self‐compassion, and also in this study, the phenomenon was associated with poor treatment results.

Another type of dysfunctional response to the diagnosis is its functionalization in the sense of a secondary gain in disease. Here, the diagnosis is used to gain attention, sympathy, support and closeness to other people (Zakka, Bitar, Lakkis, Sahar, & Koubar, 2017). Furthermore, a diagnosis can be readily accepted and functionalised as a justification for passively responding to difficult situations or passively accepting unsuccessful periods of life.

Over‐identification, functionalization as well as self‐stigmatization may all conflict with psychotherapy outcome because patients may fail to develop motivation to and hope for change. However, empirical findings are extremely limited as they are currently primarily located in stigma research.

If it is empirically shown that different coping styles influence relevant therapeutic processes, the support of functional coping could be integrated more explicitly into the education of patients about their diagnosis/psychoeducation. But due to the lack of research, there is still no instrument to operationalize different styles of coping with/processing diagnoses in patients with a diagnosis of mental disorder.

### 1.1 Aim of the present research and study design

The aim of the study was to identify styles of coping with diagnoses in patients with a diagnosis of mental disorder (preliminary qualitative study 1). Second, to develop and evaluate a related self‐report measure (study 2), called ‘Hamburger coping inventory: Questionnaire on coping with mental disorder diagnoses (CoDi)’. Third, to validate the questionnaire with a large clinical sample (study 3), to analyse specific differences between patients with different diagnoses, and finally to identify influencing factors with regard to coping styles.

Summary overview:

Step 1 Preliminary qualitative study 1

Objective: Development of the questionnaire with 59 items.
Hypothesis: There are coping styles among those affected by mental disorders that have not yet been considered in empirical research.

Step 2 Evaluation study 2 (*N =* 252 patients)

Objective: Cross‐validation of the questionnaire and development of the modified questionnaire with 26 items.
Hypothesis: A factor analytical model of different coping styles will be identified and validated in a further confirmatory analysis.

Step 3 Revaluation study 3 (*N =* 1271 patients)

Objective 1: Revaluation of the factor structure with a large sample.
Objective 2: Analyses of diagnosis‐specific coping and of further influencing factors.
Hypothesis 1: The factor analytical model can be reconfirmed in a further confirmatory analysis with a large sample of patients.
Hypothesis 2: Different copying styles can be identified for different diagnoses. Besides diagnoses, further influencing parameters may explain variance in coping styles.

All studies were carried out in compliance with the latest revision of the Declaration of Helsinki and were approved by the local ethics committee.

## 2 PRELIMINARY QUALITATIVE STUDY (STUDY 1)

### 2.1 Inclusion criteria and description of the sample

Aiming at a broad spectrum of coping styles, we included patients with various diagnoses according to DSM‐5 (American Psychiatric Association, 2013): Substance use disorder, schizophrenia, affective disorder, anxiety disorder, post‐traumatic stress disorder (PTSD), eating disorder, somatic stress disorder (SDD) and borderline personality disorder; patients had to know their diagnosis; we implicitly assumed that the length of time since a patient is confronted with a diagnosis may influence individual coping, for example, through chronification and treatment experiences. So, we included patients with varying length of time since the diagnoses were communicated; in comorbidity, one diagnosis was identified as the main diagnosis and its significance for the patient was referred to in the interview; in psychotic disorders, it was ensured that acute delusional experiences or positive formal thought disturbances (such as disorganisation) did not interfere with the capability to give informed consent.

The sample consisted of 40 (23 females and 17 males) patients (*n* = 5 for each diagnosis). Twenty five patients were in inpatient and 15 in outpatient treatment. They were between 20 and 56 years old (*M* = 35.7, *SD* = 14.5). The time passed since patients had been informed about diagnosis ranged from 2 weeks to 10 years.

### 2.2 Study procedure and data processing

Patients were recruited in surrounding psychiatric clinics and outpatient facilities. As part of the study, eight apprentice psychotherapists underwent special training in qualitative methods. They spent 3 months at disorder‐specific wards or outpatient services, recruiting patients and conducting interviews. Each of them were responsible for interviews with patients of a particular diagnosis. All patients underwent standardized initial diagnostics by means of SCID interviews (First et al., 2014). The qualitative interviews were semi‐structured using a standardized interview protocol, audiotaped, transcribed verbatim (Kuckartz, Dresing, Rädiker, & Stefer, 2008) and analysed by using qualitative content analysis (Mayring, 2010). The aim was to reduce the material into single condensed statements close to the original wording. In the next step, single statements were grouped into superior categories.

### 2.3 Construction of the questionnaire and content validation of items

A total of 59 items were derived from the patients' statements. We ensured that items were close to the original wording, linguistically simple and as specific as possible. The items related to nine categories that were taken from the qualitative analyses: (1) *Acceptance of diagnosis* (six items); (2) *Self‐acceptance*: Diagnosis experienced as a relief, increasing the acceptance of oneself (six items); (3) *Empowerment*: Diagnosis gives hope (positive change expectation), associated with the experience of self‐efficacy and motivation for change (seven items); (4) *Attribution to a higher meaning*: Diagnosis experienced as a crisis that leads to deeper reflection on oneself and the experience of inner growth (seven items); (5) *Over‐identification*: Excessive identification with the diagnosis, tendency to consciously develop symptoms, desire for demarcation from healthy people (seven items); (6) *Resignation/Hopelessness*: Firm belief that nothing can be done to recover (seven items); (7) *Self‐stigmatization*: Public stigma of diagnosis is related to one's own person (six items); (8) *Protection against strain*: The diagnosis justifies a lack of initiative for change and problem solving (six items); and (9) *Secondary gains*: Diagnosis is functionalized to ensure social attachment/attention from others (seven items).

The questionnaire was sent to five experts in the field of psychotherapy research to give feedback about the clarity of item phrasing and content validity. Both aspects were assessed per item on a 4‐point rating‐scale, ranging from ‘unclearly expressed’ or ‘unsuitable’ to ‘clearly expressed’ or ‘suitable’. Items evaluated as ‘clearly expressed’ and ‘suitable’ were retained. Items evaluated as ‘almost clearly expressed’ or ‘almost suitable’ were reworded, following suggestions of the experts. The first version of the questionnaire consisted of 59 items, each scored using a 5‐point Likert‐scale (from ‘not at all’ to ‘very much’).

The questionnaire further encompassed general questions about *socio‐demographic characteristics* (age, gender, years of education, employment statusand marital status), *psychopathology* (main diagnosis and time since diagnosed, comorbid diagnoses) and *treatment history* (months of outpatient psychotherapy and number of inpatient treatments).

## 3 EXPLORING THE FACTOR STRUCTURE (STUDY 2)

The aim of study 2 was to identify the factor structure of the questionnaire and to cross‐validate it.

### 3.1 Methods

#### 3.1.1 Inclusion criteria for sample 2

Inclusion criteria were: Age ≥18 years; German as a first language; patients were diagnosed with a mental illness according to DSM‐5 (American Psychiatric Association, 2013); patients know their diagnosis; in the case of comorbid diagnoses, one disorder is recognizable as the main diagnosis to which the patients should refer in the questionnaire (e.g., a patient with schizophrenia and comorbid depression was instructed to consider what it means for him to be diagnosed with schizophrenia in response to the questionnaire‐items); no psychotic positive symptoms or neurological disorder interfering with the ability to give informed consent.

#### 3.1.2 Procedures

Two hundred fifty‐two patients were recruited in regional clinics nearby. Diagnoses were verified using SCID interviews (First et al., 2014), conducted by apprentice psychotherapists, who were not involved in the treatment of patients. The communication of the diagnoses was explicitly not carried out within the context of the study, to represent the clinical reality in a naturalistic way, that is, both favourable and unfavourable variants of the communication. Furthermore, we included patients who had known their diagnosis for different lengths of time in order to record possible effects of different courses of the disorders, processes of chronification or treatment experiences on coping styles. After the verification of inclusion criteria, patients started to fill in the questionnaire. The entire questionnaire did not take longer than 25 min to complete.

#### 3.1.3 Statistics

Missing values were identified for eight items (min = 0.4%/max = 1.6%). The Little‐Test (Little, 1988) indicated that missing values were completely at random and therefore were imputed by the expectation‐maximization‐method. We performed a split‐half randomization with the sample to conduct a cross‐validation of the factor structure. The two sub‐samples were compared concerning demographic and clinical data (distribution of different diagnoses, the time since diagnosed with a mental disorder and the number of inpatient and length of outpatient treatments).

##### Exploratory factor analyses (sub‐sample 1, *n* = 126)

Sample adequacy and factorability of data were analysed using the Kaiser–Meyer–Olkin (KMO)‐measure and the Bartlett Test for sphericity. We performed an exploratory maximum likelihood factor analyses (EFA) with varimax rotation to analyse the best factor structure. Additionally to the scree‐test, the parallel analysis (Horn, 1965) and the Minimum‐Average‐Partial‐Test (MAP‐test) were used for determining the number of factors. Subsequently, In order to enhance homogeneity and reliability, the factor solution was further gradually optimized by means of the following criteria: Exclusion of items with content redundancy, low loadings on the main factor (<0.50), double‐loading >0.30 on a second factor, with a difference of loadings less than ±0.20 between both factors.

In addition, the following criteria were used in a *reliability analysis* for item selection:

Regarding internal consistency, Cronbach's alpha with values around 0.8 are recommended. The preferable range for item difficulty is 0.2–0.8, and good corrected item discrimination indicate indices ˃0.5 (Nunnally & Bernstein, 1994, pp. 131–147).

##### Cross‐validation through confirmatory factor analyses (sub‐sample 2, *n* = 126)

A cross‐validation of the EFA‐six‐factor solution was performed with confirmatory factor analysis (CFA) on sub‐sample 2 with *n* = 126. Assessment of overall model fit was based on multiple fit indices, including normed *χ* ^2^ minimum discrepancy, divided by its degrees of freedom (CMIN/df), Root Mean Square Error of Approximation (RMSEA), Standardized Root Mean Square Residuals (SRMR), Tucker–Lewis reliability Index (TLI) and Comparative Fit Index (CFI). A normed *χ* ^2^ of 1‐2 is considered to be an adequate fit (Byrne, 2006). Samples with *n* ≤ 250 require a RMSEA value <0.08 and for SRMR a value <0.08 (Hu & Bentler, 1999); CFI and TLI values between 0.90 and 0.95 represent acceptable to good model fit (Byrne, 1998; Hoyle, 2011). Standardized residual covariances should not exceed the interval of −2.58 ≤ *z* ≤ 2.58 (*α* = 1%; Byrne, 2010).

### 3.2 Results

#### 3.2.1 Demographic and clinical data

Table 1 compares both subsamples with regard to demographics and clinical data. No significant differences emerged.

**TABLE 1:** Demographic and clinical data of sample 2

	Descriptive data		Statistics		
	Subsample 1 (*N* = 126)	Subsample 2 (*N* = 126)	*df*	Value	*p*
Demographics					
Gender (male/female)	43/83	52/74	1	1.369^a^	0.242
Age in years (*M, SD*)	39.77 (13.6)	37.76 (12.57)	250	1.217^b^	0.225
Marital status (*N*)^c^	68/37/20/1	78/34/10/4	3	5.944^a^	0.114
Years of graduation (*M, SD*)	15.70 (4.25)	15.71 (3.54)	216	0.030^b^	0.976
Clinical data					
Time since diagnosed (months; *M, SD*)	47.67 (58.81)	51.48 (76.75)	246	−0.437^b^	0.662
Distribution of diagnoses^d^	0/14/55/34/7/16/0	4/11/57/32/6/15/1	6	5.556^a^	0.474
Number of inpatient treatments (*M, SD*)	1.69 (2.68)	1.84 (3.29)	247	−0.396^b^	0.692
Months of lifetime outpatient treatment *(M, SD*)	24.87 (39.85)	21.57 (39.55)	245	0.649^b^	0.517

Abbreviations: M, means; SD, standard deviation.

^a^ Chi‐squared Value displayed.

^b^ T‐value displayed; *p* value, level of significance; *M, SD*, mean value and standard deviation.

^c^ Single, married, divorced and widowed.

^d^ Substance use disorder/schizophrenia/affective disorders/phobic‐, obsessive compulsive‐, stress‐related‐ and somatoform disorders/eating disorders/personality disorders/hyperkinetic disorders (ADHD).

#### 3.2.2 Exploratory factor analysis

To determine the factor structure, all 59 items underwent an EFA with data of sub‐sample 1 (*n* = 126). The KMO coefficient was 0.842 and implied a good fit of the correlation matrix for factor analysis. The MSA value for 58 items was >0.50 (37 items MSA > 0.80; 14 items MSA > 0.70). The significance level was *p* < 0.001. Regarding the extraction of factors, Parallel‐Analysis and Scree‐Test supported a five to six‐factor solution, Velicer's MAP test supported seven factors. Based on a review of these solutions, six factors with a total of 26 items outperformed the other solutions with respect to clinical significance and accounted for 66.43% of the variance, more than other solutions. All items of the six‐factor solution displayed sufficient factor loadings between 0.53 and 0.96 and no item displayed content redundancy. In contrast, the rejected seven‐factor solution displayed two items with low factor loadings within factor seven (below 0.35) and another two items with content redundancy. Table 2 presents the 26 items of the CoDi, Table 3 presents the factor loadings of the final exploratory model.

**TABLE 2:** Twenty six items of the six‐factor solution

Scale	Item‐nr.	Item description
Self‐acceptance/positive clarification	8	The diagnosis helps me to accept myself
	9	Since I am aware of my diagnosis, I am able to better understand my experiences and behaviour
	11	Because of my diagnosis I better understand how my life came to be
	12	The diagnosis helps me to recognize my problems
Empowerment	13	The diagnosis helps me to realize that I can do something myself to get better
	14	The diagnosis gives me hope that it can be treated
	15	My diagnosis is a challenge in my life which I will face up to
	16	Others have overcome this diagnosis that gives me hope
Higher meaning/inner growth	53	I have grown through the confrontation with my diagnosis
	55	I think that the diagnosis has a higher meaning
	56	Since I've been dealing with the diagnosis, I am able to live more aware and intense than before
	58	Before I dealt with the diagnosis, I was more unreflective and superficial than I am now
Over‐identification	22	The diagnosis makes me special
	23	My diagnosis is ‘cool’
	24	I am proud of having this diagnosis
	25	Even though I am going to therapy, something would be missing, if I did not have the diagnosis
Self‐stigmatization	34	My diagnosis is a blemish
	35	My diagnosis makes me less valuable than others
	36	I'm ashamed of myself to other people for my diagnosis
	37	For others, I'm just the lunatic no one wants anything to do with
	38	I regard being mentally ill as a personal weakness
Secondary gains	41	If suddenly I would not have the diagnosis anymore, I would have to cope with unpleasant things
	46	I sometimes use my diagnosis to get more attention
	48	I think it is good that, due to the diagnosis, I get excused from some everyday responsibilities
	51	I think it is good that, due to my diagnosis, many decisions are being taken over by others
	52	Because of my diagnosis I can just stay in bed and let others take care of me

*Note*: The instrument was evaluated in German language. Translation was based on Brislin (1970). One bilingual student blindly translated the instrument from German to English. Another bilingual student independently back‐translated the instrument. Both versions were compared for concept equivalence, discrepancies were discussed and resolved.

**TABLE 3:** Factor loadings of the six‐factorial exploratory factor analysis (EFA)‐model

Item	Factor 1	Factor 2	Factor 3	Factor 4	Factor 5	Factor 6
8	0.54	‐	‐	‐	‐	‐
9	0.71	‐	‐	‐	‐	‐
11	0.75	‐	‐	‐	‐	‐
12	0.85	‐	‐	‐	‐	‐
13	0.36	0.59	‐	‐	‐	0.31
14	‐	0.77	‐	‐	‐	‐
15	‐	0.69	‐	‐	‐	‐
16	‐	0.75	‐	−0.31	‐	‐
22	‐	‐	0.73	‐	‐	‐
23	‐	‐	0.95	‐	‐	‐
24	‐	‐	0.96	‐	‐	‐
25	‐	‐	0.72	‐	‐	‐
34	‐	‐	‐	0.76	‐	‐
35	‐	‐	‐	0.80	‐	‐
36	‐	‐	‐	0.82	‐	‐
37	‐	‐	‐	0.63	‐	‐
38	‐	‐	‐	0.67	‐	‐
41	‐	‐	‐	‐	0.55	‐
46	‐	‐	‐	‐	0.53	‐
48	‐	‐	‐	‐	0.79	‐
51	‐	‐	‐	‐	0.87	‐
52	‐	−0.36	‐	‐	0.68	‐
53	‐	‐	‐	−0.32	‐	0.71
55	‐	‐	‐	−0.34	‐	0.57
56	‐	‐	‐	−0.40	‐	0.69
58	‐	‐	‐	‐	‐	0.67

*Note*: Data refer to subsample 1 of sample 2; explained variance: 66.43 %; Factor 1 = Self‐acceptance/positive clarification; Factor 2 = Empowerment; Factor 3 = Over‐identification; Factor 4 = Self‐stigmatization; Factor 5 = Secondary gains; Factor 6 = Higher meaning/inner growth.

#### 3.2.3 Confirmatory factor analyses

CFA using data of sub‐sample 2 (*n* = 126) confirmed the EFA solution. All items loaded high on their respective factor with *p* < 0.001. However, a Heywood case (Kline, 2011, p. 158) occurred for item 23, whose standardized regression‐weight was >1 (*λ* = 1.01). According to Kolenikov and Bollen (2012), false specifications are the most common causes of negative variance estimates. The Mardia‐test showed a significant violation of the normal distribution for items 23 and 24 of the scale ‘over‐identification’ (items listed in Table 2). Both items revealed skewness and kurtosis since these items are probably negated by most patients with depression who are overrepresented in the sample. Following Kline (2011), the problem was solved by releasing the parameter for the covariance of the error terms of both items. There were no significant deviations from the standardized residual covariance‐matrix. Standardized residuals were within the proposed significance range (−2.58 ≤ *ᴢ* ≤ 2.58) according to Byrne (2010).

**FIGURE 1:**
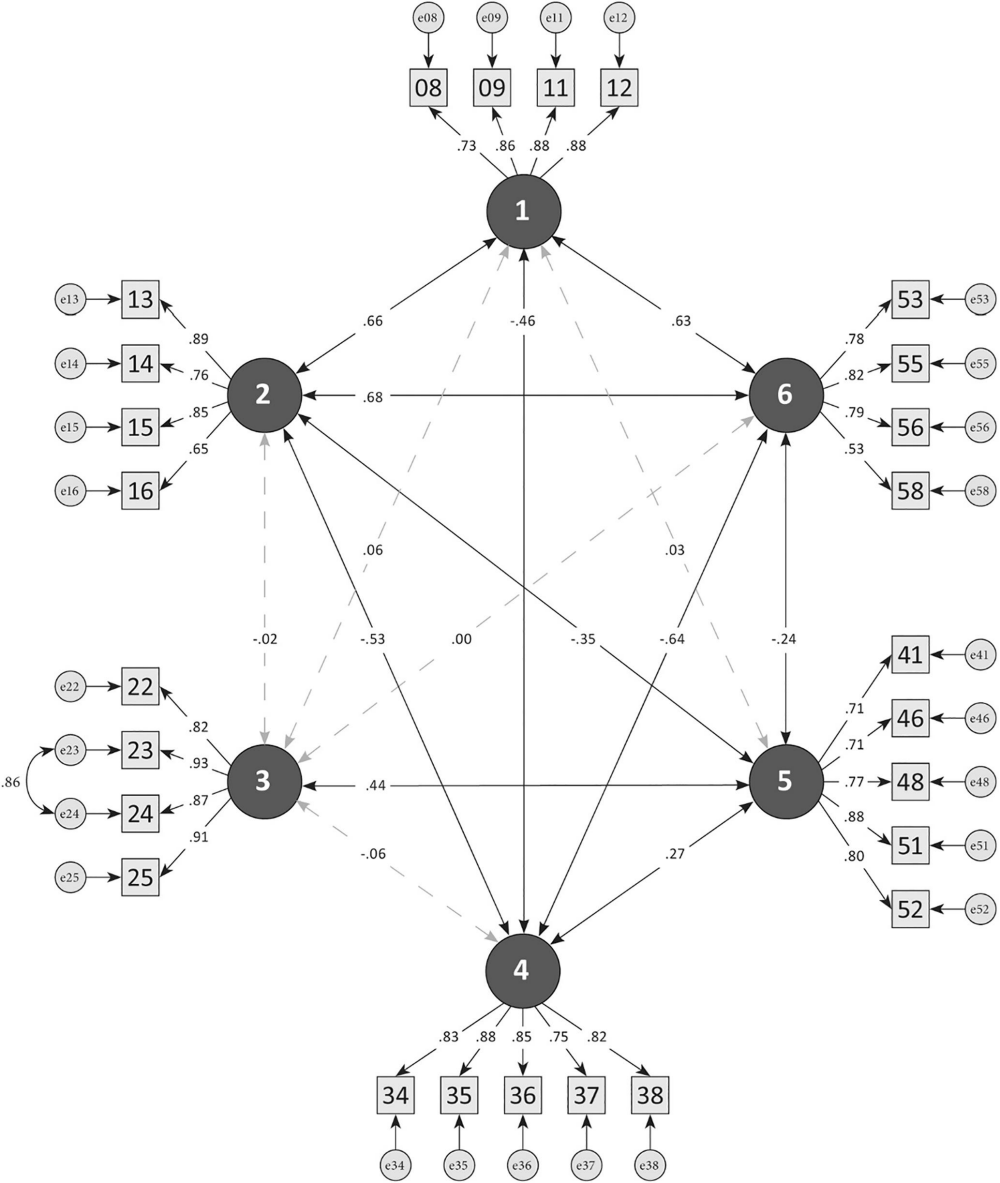
Path model. Dashed lines = Factor‐correlations < 0.10; Factor 1 = Self‐acceptance/Positive clarification; Factor 2 = Empowerment; Factor 3 = Over‐Identification; Factor 4 = Self‐Stigmatization; Factor 5 = Secondary Gain; Factor 6 = Higher Meaning/Inner Growth

The chi‐squared test calculated a significant result (*χ* ^2^(288) = 447.793; *p* < 0.001). The normed chi‐squared value of 1.555 outlined an adequate model fit. The RMSEA‐value of 0.067 was within the required range for small samples. The SRMR*‐*value of 0.0688 displayed a good model fit, and the CFI value of 0.936 and TLI value of 0.927 reached the limit of 0.90 to represent an acceptable model fit. Figure 1 presents the corresponding path‐model.

#### 3.2.4 Psychometric characteristics of items and scales

All values were within an acceptable range: Item difficulties ranged between 0.26 and 0.80; discrimination indices ranged between 0.53 and 0.88; alpha coefficients of factors ranged between 0.84 (factor 5) and 0.90 (factor 3). Table 4 outlines the results of the reliability analyses, including mean values and standard deviations of the scales.

**TABLE 4:** Factor mean values and standard deviations, Item difficulty, discrimination and alpha coefficients

Factor	M (SD)	Item	Difficulty	Discrimination	Alpha coefficients
1	3.69 (0.96)	8	0.68	0.641	‐
	‐	9	0.76	0.750	‐
	‐	11	0.76	0.720	‐
	‐	12	0.77	0.779	‐
Factor 1 total	‐	‐	0.74 (min: 0.68/max: 0.77)	‐	0.867^a^
2	3.81 (0.97)	13	0.79	0.739	‐
	‐	14	0.77	0.833	‐
	‐	15	0.80	0.763	‐
	‐	16	0.73	0.742	‐
Factor 2 total	‐	‐	0.77 (min: 0.73/max: 0.80)	‐	0.895^a^
3	1.96 (0.91)	22	0.34	0.722	‐
	‐	23	0.26	0.870	‐
	‐	24	0.28	0.881	‐
	‐	25	0.32	0.699	‐
Factor 3 total	‐	‐	0.30 (min: 0.26/max: 0.34)	‐	0.905^a^
4	2.46 (1.16)	34	0.54	0.750	‐
	‐	35	0.46	0.837	‐
	‐	36	0.53	0.800	‐
	‐	37	0.39	0.684	‐
	‐	38	0.52	0.694	‐
Factor 4 total	‐	‐	0.49 (min: 0.39/max: 0.54)	‐	0.900^a^
5	1.82 (0.92)	41	0.38	0.549	‐
	‐	46	0.35	0.530	‐
	‐	48	0.37	0.699	‐
	‐	51	0.33	0.766	‐
	‐	52	0.33	0.668	‐
Factor 5 total	‐	‐	0.35 (min: 0.33/max: 0.38)	‐	0.838^a^
6	3.34 (1.11)	53	0.73	0.767	‐
	‐	55	0.72	0.691	‐
	‐	56	0.68	0.818	‐
	‐	58	0.61	0.607	‐
Factor 6 total	‐	‐	0.69 (min: 0.61/max: 0.73)	‐	0.866^a^

*Notes*: *N* = 252. M (SD) = mean value and standard deviation. Factor 1 = Self‐acceptance/positive clarification; Factor 2 = Empowerment; Factor 3 = Over‐identification; Factor 4 = Self‐stigmatization; Factor 5 = Secondary gains; Factor 6 = Higher meaning/inner growth; With three items, their removal would improve the alpha of their corresponding scale (Item 8, Item 25, Item 58). However, they were retained to better reflect the content width of the dimension and to ensure at least 4 items per scale; The ‘difficulties’ of the items differ strongly depending on the scale. In particular, ‘over‐identification’ has relatively lower values because the diagnoses (especially anorexia nervosa) for which the phenomenon is known are underrepresented in our sample.

^a^ Cronbach's alpha.

Inspection of the intercorrelations of the scales showed that the three functional coping styles correlated positively with each other and negatively with self‐stigmatization (each by 0.5). Over‐identification correlated positively with functionalization with *r* = 0.443. According to Cohen, Cohen, West, and Aiken (2003), the scales are thus considered sufficiently independent of each other.

## 4 FURTHER VALIDATION (STUDY 3)

The aim of study 3 was first to test the factor structure of the CoDi with a large independent sample. Second, we analysed diagnoses specific differences on coping styles. Finally, we analysed the impact of potentially influencing factors such as *age*, *employment status, the time since diagnosed with the mental disorder, months of outpatient treatment* and the *number of inpatient treatments*, following Werner et al. (2008) and Adewuya et al. (2011).

### 4.1 Methods

#### 4.1.1 Inclusion criteria for sample 3

The inclusion criteria were identical to those in study 2.

#### 4.1.2 Procedures

CoDi with 26 items resulting from study 2 was used in the following 12 months by our apprentice psychotherapists during practice time in psychiatric hospitals. They included a total of 1.271 patients (69% inpatient, 31% outpatient), who filled in the CoDi. As in study 2, diagnoses were verified using standardized clinical interviews.

#### 4.1.3 Statistics

##### Confirmatory factor analyses

The larger sample size required stricter parameters for good model fit (Schermelleh‐Engel, Moosbrugger, & Müller, 2003), such as RMSEA < 0.06, SRMR < 0.05, CFI, TLI as well as normed fit index (NFI) and incremental fit index (IFI) between 0.90 and 0.95 (Byrne, 1998; Hoyle, 2011; Hu & Bentler, 1999). Missing values were identified for five items (min = 0.3%/max = 1.8%). We used multiple imputation to accommodate missing data by the expectation‐maximization‐method.

##### Group differences between diagnoses and analysis of further influencing factors

Group differences between diagnoses with regard to coping styles and impact of further potential influencing factors were analysed by means of a multivariate analysis of covariance (MANCOVA). Four diagnoses were excluded from analyses because of small sample sizes (Bulimia nervosa, *n* = 17; bipolar affective disorder, *n* = 16; ADHD, *n* = 13; and autism spectrum disorder, *n* = 3). Diagnoses (nine categories) and employment status (two categories: yes vs. no) were independent variables. Age and the number of inpatient treatments were included as covariates.

Post hoc analyses were applied to specify main and interaction effects. Lastly, we performed a post hoc power analysis, using the program ‘G‐Power 3.1.9.2’ (Faul, Erdfelder, Buchner, & Lang, 2009). Statistical power (1‐β error probability) was computed as a function of significance level, sample size and population effect size. Values over 0.8 report sufficient power. Effect sizes of the partial *η* ^2^ are reported. The level of significance was adjusted to 5%. Corrections were made according to Bonferroni. All procedures were performed using SPSS (SPSS Inc.) and SPSS‐AMOS (Version 25.0).

### 4.2 Results

#### 4.2.1 Demographic and clinical data

Sample 3 (*n* = 1271) consisted of 825 (64.8%) female and 446 (35%) male patients. Mean age was 36.6 years (*SD* = 13.2). 64.6% of patients were single, 22.1% were married, 11.6% were divorced and 1.7% were widowed. 581 (45.6%) patients were employed, 690 (54.1%) were unemployed. An average of 51.2 months ago (*SD* = 67.4) they were diagnosed with a mental disorder and received an average of three inpatient psychiatric treatments (*SD* = 6). 54.1% of the patients had a comorbid disorder, 19% suffered from a second comorbidity. Frequencies of the main diagnoses, employment status and mean values regarding coping styles are displayed in Table 5.

**TABLE 5:** Frequencies of main diagnoses in sample 3 (*N* = 1.271), employment status within each diagnose and means (standard deviations) within diagnoses with regard to coping styles

	Diagnoses												Employment	
	SUD	Sz	Dep	Bip^a^	Anx	PTSD	SSD	AN	BN^a^	BPD	ADD^a^	ASS^a^	Yes	No
*N* (%)	142 (11.2)	114 (9.0)	427 (33.6)	16 (1.3)	128 (10.1)	95 (7.5)	30 (2.3)	42 (3.3)	17 (1.3)	244 (19.2)	13 (1.0)	3 (0.2)	581 (45.6)	690 (54.1)
Clarific. *M (SD)*	3.63 (0.91)	3.13^d^ (1.13)	3.83 (0.77)	3.59 (0.64)	3.83 (0.89)	3.88^c^ (0.65)	3.33 (1.21)	2.82^e^ (1.37)	3.55 (1.0)	3.6 (1.05)	3.8 (0.6)	4.25 (0.86)	3.83 (0.81)	3.5 (1.04)
Empow. *M (SD)*	3.93 (0.78)	3.23^h^ (1.09)	3.94^f^ (0.73)	3.19 (0.84)	4.07^g^ (0.76)	3.84 (0.67)	3.06 (1.5)	2.62^i^ (1.45)	4.02 (0.76)	3.43 (1.04)	3.23 (0.9)	2.83 (0.8)	3.94 (0.83)	3.51 (1.01)
Growth *M (SD)*	3.18 (0.96)	2.7^k^ (1.15)	3.42 (0.86)	3.21 (0.78)	3.25 (0.93)	3.52^j^ (0.92)	2.84 (1.3)	3.13 (1.2)	3.44 (0.85)	3.05 (1.01)	3.15 (0.78)	3.16 (0.87)	3.4 (0.9)	3.06 (1.04)
Over‐Id. *M (SD)*	1.66 (0.7)	1.6 (0.86)	1.56 (0.71)	1.62 (1.05)	1.44 (0.62)	1.41 (0.67)	1.55 (0.83)	4.1^l^ (1.33)	2.2 (1.6)	3.12^m^ (1.03)	2.07 (0.8)	1.75 (0.66)	1.58 (0.74)	1.86 (1.09)
Stigmat. *M (SD)*	2.91 (0.98)	3.4^n^ (1.1)	2.37 (1.03)	2.61 (0.92)	2.54 (0.99)	2.54 (1.1)	2.72 (1.16)	2.09 (1.26)	2.18 (1.11)	3.15^o^ (1.16)	2.38 (1.0)	3.06 (1.1)	2.46 (1.04)	2.88 (1.16)
Gains *M (SD)*	1.85 (0.73)	2.24 (1.01)	1.82 (0.74)	2.12 (1.0)	1.64 (0.7)	1.8 (0.77)	2.8^p^ (1.33)	2.6^q^ (1.42)	1.51 (0.96)	1.99 (0.87)	1.73 (0.72)	1.53 (0.57)	1.75 (0.75)	2.06 (0.94)
Employ. yes/no^b^	48/94	30/84	236/191	8/8	80/48	48/47	16/14	11/31	9/8	86/158	8/5	1/2	‐	‐

Abbreviations: ADD, attention deficit and hyperactivity disorder; AN, anorexia nervosa; Anx, anxiety and obsessive compulsive disorder; ASS, autism spectrum disorder; Bip, bipolar psychoses; BN, bulimia nervosa; BPD, borderline personality disorder; Clarific., Self‐acceptance/positive clarification; Dep, depression; Empow., Empowerment; Gains, Secondary Gains; Growth, Higher Meaning/Inner Growth; Over‐Id., Over‐Identification; PTSD, posttraumatic stress disorder; SD, standard deviation; SSD, somatic stress disorder; Stigmat., Self‐Stigmatization; SUD, substance use disorder; Sz, schizophrenia.

^a^ Diagnoses excluded from analysis of variance due to low numbers of patients; Employ., Employment.

^b^ Frequencies; M (SD), mean value and standard deviation.

^c^ Differs from Sz (*p* ≤ 0.001), SSD (*p* = 0.040), AN (*p* = 0.002).

^d^ Differs from PTSD (s.a.), SUD (*p* = 0.035), Dep (*p* ≤ 0.001), Anx (*p* = 0.002).

^e^ Differs from PTSD (s.a.), Dep (*p* = 0.005), Anx (*p* = 0.021), BPD (*p* = 0.016).

^f^ Differs from Sz, AN, SSD, BPD (all *p* ≤ 0.001).

^g^ Differs from AN, BPD, Sz, SUD (all *p* ≤ 0.001).

^h^ Differs from SUD, Dep, Anx (all *p* ≤ 0.001), PTSD (*p* = 0.003).

^i^ Differs from Anx, SUD, Dep, PTSD (all *p* ≤ 0.001), BPD (*p* = 0.038).

^j^ Differs from Sz (*p* ≤ 0.001), SSD (*p* = 0.013), *BPD* (*p* = 0.026).

^k^ Differs from Dep, PTSD (all *p* ≤ 0.001).

^l^ Differs from all other diagnoses (all *p* ≤ 0.001).

^m^ Differs from An, Dep, Anx, PTSD (all *p* ≤ 0.001).

^n^ Differs from all other diagnoses (all *p* ≤ 0.001).

^o^ Differs from Dep (*p* ≤ 0.001), *PTSD* (*p* = 0.020), AN (*p* = 0.007).

^p^ Differs from of all other diagnostic categories (all *p* ≤ 0.001) except for Sz and AN.

^q^ Differs from all other diagnostic categories (all *p* between 0.001 and 0.022) except for Sz and SSD.

#### 4.2.2 CFA with data of sample 3

The six‐factor solution was subjected to another CFA with the data of sample 3. Parameters of model fit proved to be overall good and confirmed the dimensional structure of the CoDi, requiring no further modifications (RMSEA = 0.051; SRMR = 0.048; CFI = 0.943; TLI = 0.935; IFI = 0.943; NFI = 0.927 and RFI = 0.917).

#### 4.2.3 Results of the MANCOVA

There was a medium significant main effect for the diagnostic categories (*F* = 9733; *p* ≤ 0.001; *df* = 48/7368; *partial η* ^*2*^ = 0.069), a small to medium main effect for employment status (*F* = 9610; *p* ≤ 0.001; *df* = 6/1223; *partial η* ^*2*^ = 0.045), a small main effect for age (*F* = 7.060; *p* ≤ 0.001; *df* = 6/1223; *partial η* ^*2*^ = 0.033) and the number of inpatient treatments (*F* = 5.077; *p* ≤ 0.001; *df* = 6/1223; *partial η* ^*2*^ = 0.024). In addition, a small interaction effect (diagnoses *x* employment) was significant (*F* = 2.320; *p* ≤ 0.001; *df* = 48/7368; *partial* ^*2*^ = 0.025). Results of the post hoc power analyses for *F*‐tests revealed excellent statistical power. All values ranged between 0.91 and 0.99.

Post hoc ANOVAs provided various significant results. However, in some cases the effect sizes were below the limit for small effects according to Cohen (1988; <0.010). In the following, only at least small effect sizes were reported. No effects were identified for the past time since diagnosed with the mental disorders and for the months of outpatient treatment.

##### Effects for the variable ‘diagnoses’

A large effect was evident for *over‐identification* (*F* = 26.394; *p* ≤ 0.001; *df* = 8; *partial η* ^*2*^ = 0.147). Medium effects were identified for *empowerment* (*F* = 12.086; *p* ≤ 0.001; *df* = 8; *partial η* ^*2*^ = 0.075) and for *self‐stigmatization* (*F* = 8.887; *p* ≤ 0.001; *df* = 8; *partial η* ^*2*^ = 0.055). Small to medium effects were identified for *self‐acceptance/positive clarification* (*F* = 6.207; *p* ≤ 0.001; *df* = 8; *partial η* ^*2*^ = 0.041), *secondary gains* (*F* = 6.466; *p* ≤ 0.001; *df* = 8; *partial η* ^*2*^ = 0.040) and *higher meaning/inner growth* (*F* = 5.259; *p* ≤ 0.001; *df* = 8; *partial η* ^*2*^ = 0.035).

Pairwise comparisons of diagnoses identified specific diagnoses for each style of coping that differ significantly from certain other diagnoses. Particularly interesting was that *self‐acceptance/positive clarification* as well as *higher meaning/inner growth* were particularly pronounced in *PTSD*, whereas *over‐identification* was particularly pronounced in *AN,* followed by *Borderline personality disorder. Self‐stigmatization* was pronounced in *schizophrenia* and s*econdary gains* was pronounced in *SDD,* followed by *AN* (see Table 5 for superscripts that indicate all significant differences and *p*‐values in the legend).

##### Effects for ‘employment’

There were small effects regarding *empowerment* (*F* = 26.346; *p* ≤ 0.001; *df* = 1; *partial η* ^*2*^ = 0.021) and *over‐identification* (*F* = 26.881; *p* ≤ 0.001; *df* = 1; *partial η* ^*2*^ = 0.021). Inspection of descriptive data from Table 5 indicates that unemployment is associated with lower empowerment and higher over‐identification with the diagnosis.

##### Effects for ‘number of inpatient treatments’

There is a small to medium effect regarding *self‐stigmatization* (*F* = 24.371; *p* ≤ 0.001; *df* = 1; *partial η* ^*2*^ = 0.029). Self‐stigmatization is more pronounced with a higher number of inpatient treatments.

##### Effects for ‘age’

There were just very small effects regarding *self‐acceptance/positive clarification* (*F* = 16.730; *p* ≤ 0.001; *df* = 1; *partial η* ^*2*^ = 0.013) and *over‐identification* (*F* = 16.107; *p* ≤ 0.001; *df* = 1; *partial η* ^*2*^ = 0.013). Younger age is associated with lower empowerment and higher over‐identification with the diagnosis.

##### Effects for the interaction ‘diagnoses × employment’

There were small to medium effects regarding *over‐identification* (*F* = 5.892; *p* ≤ 0.001; *df* = 8; *partial η* ^*2*^ = 0.037) and *self‐stigmatization* (*F* = 2.626; *p* = 0.007; *df* = 8; *partial η* ^*2*^ = 0.019).

## 5 DISCUSSION

We developed CoDi, a reliable standardized self‐report measure on the consequences of informing patients about their diagnoses of mental disorders. We pursued the hypothesis that there are diagnosis‐related specifics in this regard. CoDi with good model fit consists of three functional (positive clarification/self‐acceptance, empowerment and higher meaning/inner growth) and three dysfunctional (self‐stigmatization, over‐identification and secondary gains) styles of coping with diagnoses. Diagnoses differ in presenting these styles with medium effect sizes. In addition, an interaction between employment status and diagnosis revealed medium effect sizes with regard to coping types. Small effects were seen for unemployment and the number of inpatient treatments, both associated with unfavourable coping.

‘*Positive clarification/self‐acceptance*’ as well as ‘*empowerment*’ is theoretically related to the model of SDM. The diagnosis can heighten the patients' self‐esteem and give them the strength to adapt to the situation (Craddock & Mynorss‐Wallis, 2014). It is further known that SDM affects relevant treatment processes (Joosten et al., 2008). Accordingly, inspection of the literature revealed hypothetical associations between functional coping and well known common factors of psychotherapy. *Positive clarification/self‐acceptance* is theoretically related to the common factor ‘*motivational clarification*’ (Grawe, 2004). Both focus on a better understanding of oneself and the significance of the disease for one's own development. Empowerment refers to hope for change and self‐efficacy, associated with the common factors ‘*expectation of therapeutic success*’ (Weinberger, 1995) and ‘*resource activation*’ (Grawe, 2004). The third processing style (*higher meaning/internal growth*) is associated with Mazor, Gelkopf, and Roe (2018), emphasizing that psychiatric diagnosis can be processed almost traumatically, which in turn could generate post‐traumatic inner growth after the crisis has been overcome. Posttraumatic growth is defined as experience of positive change that occurs as a result of the struggle with challenging crises (Tedeschi & Calhoun, 2004). Especially patients with PTSD are associated with that. Accordingly, in the present study, PTSD is the only diagnosis that differs significantly from others in this dimension. Further, there are associations with the common factor a new narrative about the self and ones life history (Jorgensen, 2004). Subsequent prospective studies may clarify the relationship between types of coping and common factors.

‘*Self‐stigmatization*’ represents a typical and frequent obstacle in patients with mental disorders. Empirical support especially exists for schizophrenia, but also for substance use disorders (SuD) and borderline personality disorders (BPD; Feldhaus et al., 2018). It is therefore consistent that in the present study schizophrenia patients differed significantly from other diagnoses in terms of self‐stigmatization, except for SuD and BPD. Self‐stigmatization triggers hopelessness, conflicting with therapeutic change agents and adherence to treatment (Feldhaus et al., 2018). Studies indicate accordingly that self‐stigmatization in patients is a hindrance to recovery (Surmann et al., 2017) and associated with longer duration of mental disorders (Adewuya et al., 2011). This fits with the present finding that self‐stigmatization increases with the *number of inpatient treatments*. The hope for recovery may be reduced due to repeated treatment, if results are not sustainable. Correlations between the coping styles can be understood in terms of content, especially with regard to self‐stigmatisation and empowerment. This has already been confirmed by other studies, for example by Zhang et al. (2017), where empowerment was positively and self‐stigma negatively associated with treatment outcome.

‘*Over‐identification*’ refers to an excessive identification of patients with the diagnosis. This phenomenon seems quite diagnoses‐specific. In particular, empirical support for an over‐identification just refers to patients with AN (Sunkel, 2015). This is also reflected in the present data, as patients with AN were significantly more over‐identified with their diagnosis than other patients. In addition to AN, in the present study, patients with BPD were significantly more over‐identified with their diagnosis compared to other patients (apart from AN). Uncertainty about one's own identity could lead to willing adoption of the diagnosis to clarify the experience of identity, and both diagnoses are associated with problematic identity development: In BPD, uncertainty about ones own identity is a core criterion for diagnostics. Regarding AN, a multifactorial etiology is assumed (Sibeoni et al., 2017). However, since the disease develops primarily in young women, often in the context of pubertal changes, it can be postulated that uncertainty about one's own identity plays a significant role (Zeeck, 2018). Additionally, the *employment status* as well as the interaction (*employment* × *diagnoses)* explained variance regarding over‐identification. The interaction effect may refer to the diagnoses‐specific character of over‐identification. And since employment is a source of identity for many people (Luyckx, Schwartz, Goossens, & Pollock, 2008), the hypothesis can be made that the lack of identification with a meaningful job increases the risk of dysfunctional identification with one's diagnosis. In particular, this could be imagined for diagnoses whose psychopathology is associated with a disturbance in the experience of identity, such as BPD and AN.

When patients *functionalize* diagnoses in order to gain certain benefits (social attachment, attentiveness), it is described as ‘*secondary gains*’. Although there is no systematic research, practitioners fear this phenomenon because it contradicts any motivation for change in patients. In particular, patients with SDD have a reputation for functionalizing their diagnosis and adopting a more passively and demanding attitude towards their therapist (Sachse, 2018). Present data fit this description, as the diagnosis SSD differed significantly from others. To summarize, the diagnosis‐specific findings in the present study, as well as the fact that the factor ‘diagnoses’ brought the highest variance explanation, indicates the validity of the present construct.

Follow‐up studies should identify parameters that explain different coping in patients with different diagnoses and should further clarify, to what extent different coping influences relevant processes and outcome of therapy. This could be important in the diagnostic information dialogue, but also beyond that. The inner attitude therapists take towards the psychopathology or diagnosis of patients most likely influences the way patients cope with their diagnosis and develop in therapy (Horsfall, Cleary, & Hunt, 2010), following the looping effect of Hacking (2007), in that ‘people who are classified in a certain way tend to grow into the way they are described’. Using the common medical model, mental disorders are to be described as successfully treatable diseases of the brain. This approach has proven to be useful in reducing blame in relation to mental illness (Corrigan, Watson, Byrne, & Davis, 2005). Unfortunately, such messages can also exacerbate stigma by reinforcing notions of individual differences and defects (Corrigan et al., 2005). Various studies indicate that stigmatising attitudes towards mental disorders can also be found among health care professionals (Kopera et al., 2015; Reavley, Mackinnon, Morgan, & Jorm, 2014). And with regard to self‐stigmatization, well‐founded research exists, indicating negative treatment‐relevant effects: individuals with mental disorders who stigmatize themselves tend to avoid psychiatric treatment (Vogel, Wade, & Haake, 2006), and self‐stigmatization negatively influences the efficacy of treatment (Ociskova et al., 2014). Conversely, empowerment is associated with recovery (Zhang et al., 2017). The intuitively plausible assumption that other coping styles are also of relevance regarding the course of mental disorders should be analysed in further studies. For example, it is intuitively plausible that over‐identification with the diagnosis and functionalisation tends to motivate affected patients to stick to their diagnosis rather than to develop motivation for treatment and change.

Besides the medical model, another approach of communicating diagnoses has gained acceptance in psychoeducation for some diagnoses. It focuses on the normalization and depathologization of symptoms, and in PTSD, this approach had a beneficial effect on self‐stigmatization and may support empowerment of patients (Hundt, Robinson, Arney, Stanley, & Cully, 2015). In American borderline therapy studies, moreover, very specific therapists proved to be particularly successful (‘super shrinks’) who did not pathologize their patients but saw them as partners in a solidary fight against difficult living conditions (Gunderson et al., 2003). It can be assumed that a normalizing attitude of the therapists had a favourable effect on the risk of self‐stigmatisation and over‐identification.

In summary, a complex system of different influencing parameters seems to have an effect on the coping of patients, which in turn regulates therapeutically relevant processes and their outcome.

### 5.1 Limitations

Parallel analysis, Velicer's MAP test and scree‐test provided different factor solutions (five, six or seven factors). The six factor solution represented the object better than five or seven factor solutions for content reasons, and one factor of the seven factor solution only consisted of two items. The ‘difficulty’ indices of the items differ strongly depending on the scales, which is due to the fact that some of the phenomena are very diagnosis‐specific. Accordingly, the inhomogeneous distribution of the different diagnoses may have led to some factors being overestimated and others underestimated. For future studies, this may also be accompanied by deviations from the normal distribution. Effect sizes of the variance analysis were small in many domains. So, it should be considered that there are potential influencing variables which have not been identified in the present study, such as possible gender effects as well as the possible impact of the therapeutic alliance on how patients cope with their diagnoses. The present research is further based on cross‐sectional data only. Longitudinal studies would allow a more rigorous investigation of the stability of the factor structure as well as antecedents and consequences of the coping styles. In addition, reanalyses should examine the existing factor structure, also with regard to possible higher order factors. Finally, this questionnaire was validated using data from German patients only. Therefore, further studies are needed for validating the translated items for the English speaking population.

### 5.2 Conclusions

In summary, CoDi is a novel questionnaire on the consequences of informing patients about their diagnoses with sound psychometric properties. It is the first instrument that enables the operationalization of functional and dysfunctional variants of how patients cope with diagnosis. Specific diagnosis‐dependent effects could be identified that should be considered in the treatment of patients. Future prospective longitudinal studies may consider the extent to which different types of coping influence therapy processes such as common factors and outcome measures.

## ARTICLE FUNDING

Open access funding enabled and organized by Projekt DEAL.

## CONFLICT OF INTEREST

The authors report no conflict of interest.

## AUTHOR CONTRIBUTIONS

Thomas Schnell contributed to conception and design of the study, coordinated the study and wrote the manuscript. Anja Kehring recruited data and contributed to statistical analyses. Olaf Morgenroth supervised statistical analyses. Steffen Moritz contributed to the interpretation of the results and has been involved in revising the manuscript. All authors read and approved the final manuscript.
